# Performance Projection of Vacuum Gate Dielectric Doping-Free Carbon Nanoribbon/Nanotube Field-Effect Transistors for Radiation-Immune Nanoelectronics

**DOI:** 10.3390/nano14110962

**Published:** 2024-06-01

**Authors:** Khalil Tamersit, Abdellah Kouzou, José Rodriguez, Mohamed Abdelrahem

**Affiliations:** 1 National School of Nanoscience and Nanotechnology, Abdelhafid Ihaddaden Science and Technology Hub, Sidi Abdellah, Algiers 16000, Algeria; 2 Department of Electronics and Telecommunications, Université 8 Mai 1945 Guelma, Guelma 24000, Algeria; 3 Laboratory of Inverse Problems, Modeling, Information and Systems (PIMIS), Université 8 Mai 1945 Guelma, Guelma 24000, Algeria; 4 Applied Automation and Industrial Diagnosis Laboratory (LAADI), Faculty of Science and Technology, Djelfa University, Djelfa 17000, Algeria; kouzouabdellah@ieee.org; 5 Electrical and Electronics Engineering Department, Nisantasi University, Istanbul 34398, Turkey; 6 High-Power Converter Systems (HLU), Technical University of Munich (TUM), 80333 Munich, Germany; 7 Center for Energy Transition, Universidad San Sebastián, Santiago 8420524, Chile; jose.rodriguezp@uss.cl; 8 Electrical Engineering Department, Faculty of Engineering, Assiut University, Assiut 71516, Egypt

**Keywords:** vacuum, radiation hardness, carbon nanotube (CNT), graphene nanoribbon (GNR), field-effect transistor (FET), tunnel FET (TFET), non-equilibrium Green’s function (NEGF), quantum simulation, doping-free

## Abstract

This paper investigates the performance of vacuum gate dielectric doping-free carbon nanotube/nanoribbon field-effect transistors (VGD-DL CNT/GNRFETs) via computational analysis employing a quantum simulation approach. The methodology integrates the self-consistent solution of the Poisson solver with the mode space non-equilibrium Green’s function (NEGF) in the ballistic limit. Adopting the vacuum gate dielectric (VGD) paradigm ensures radiation-hardened functionality while avoiding radiation-induced trapped charge mechanisms, while the doping-free paradigm facilitates fabrication flexibility by avoiding the realization of a sharp doping gradient in the nanoscale regime. Electrostatic doping of the nanodevices is achieved via source and drain doping gates. The simulations encompass MOSFET and tunnel FET (TFET) modes. The numerical investigation comprehensively examines potential distribution, transfer characteristics, subthreshold swing, leakage current, on-state current, current ratio, and scaling capability. Results demonstrate the robustness of vacuum nanodevices for high-performance, radiation-hardened switching applications. Furthermore, a proposal for extrinsic enhancement via doping gate voltage adjustment to optimize band diagrams and improve switching performance at ultra-scaled regimes is successfully presented. These findings underscore the potential of vacuum gate dielectric carbon-based nanotransistors for ultrascaled, high-performance, energy-efficient, and radiation-immune nanoelectronics.

## 1. Introduction

Radiation-sensitive field-effect transistors (RADFETs) serve as indispensable sensors for radiation monitoring and sensing, providing critical data for ensuring safety across various industries and domains [1,2,3]. Their unique functionality enables high-performance and efficient detection and measurement of radiation levels and rates, making them practical tools in military and defense, medicine (including radiotherapy and radiation dosimetry), space exploration, and high-energy physics experiments [4,5,6]. These field-effect transistors feature a sensitive gate oxide that, when exposed to radiation under some biasing conditions, traps charges within the oxide layer and/or interfaces. This results in a change in the transfer characteristics of the field-effect transistors, including the threshold voltage [1,2,3,4,5,6]. In this context, the use of an appropriate readout circuit to track these electrical changes enables accurate measurement of radiation dose [7,8].

In fact, any field-effect transistor can operate as a radiation-sensitive field-effect transistor if its gate dielectric is radiation-sensitive, with some considerations in terms of biasing conditions and sensing principles [9]. However, using FETs for switching and digital applications within radioactive environments presents challenges, mainly due to the radiation-induced threshold voltage shift mentioned earlier [10,11,12]. This shift can profoundly affect device performance, impacting parameters such as speed, power consumption, and noise margins, potentially resulting in fluctuations in system functionality [9,10,11,12,13]. In aerospace applications, these radiation-induced electrical issues can lead to serious consequences, including long-term reliability concerns, loss of control, communication breakdown, and even mission failure [14,15,16,17]. Radiation shielding using a specific metal chassis is a prevalent solution to mitigate the effects of radiation on electronic components, including field-effect transistors, in aerospace and other radiation-intensive environments [18]. The metal chassis acts as a barrier, absorbing or deflecting incoming radiation and minimizing its impact on sensitive electronics [18,19]. However, employing a metal radiation shield can significantly increase the weight of the relevant equipment, thereby impacting the overall launch cost. Even with the utilization of advanced materials to address the weight-immunity trade-off, cost remains a primary concern [18,19]. Another innovative solution is the use of FETs based on the vacuum paradigm [20,21,22,23]. The fundamental concept behind radiation-immune vacuum devices involves eliminating the local cause of radiation-induced effects, which is the gate oxide and/or channel [20,21,22,23,24,25,26]. In other words, the immunity of vacuum devices to radiation stems from the absence of a semiconductor channel and/or dielectric material, where radiation-induced multi-defects occur. The vacuum FETs have shown intriguing and promising performance in terms of immunity to radiation, electrical and switching behavior, and miniaturization [20,21,22,23,24,25,26,27,28,29].

On the other hand, utilizing carbon nanotubes (CNTs) and graphene nanoribbons (GNRs) as channels in FETs provides advantages, including elevated carrier mobility, decreased susceptibility to defects, and heightened mechanical flexibility. Particularly, the low surface-to-volume ratio of CNTs and GNRs significantly minimizes the interaction between radiation and the channel, reducing the probability of radiation-induced effects [30,31,32,33]. These materials facilitate the creation of compact, high-efficiency devices with enhanced resistance to radiation, rendering them well-suited for a wide array of electronic applications, including radiation-immune nanoelectronics [30,31,32,33]. In this context, the combination of carbon-based FET channels with the vacuum paradigm can create intriguing vacuum devices with high performance and superior immunity to radiation.

In light of the above technological developments, this paper investigates the performance of new proposed vacuum gate dielectric doping-free carbon nanotube/nanoribbon field-effect transistors (VGD-DL CNT/GNRFETs) using computational methods. We employ a quantum simulation approach that solves the Poisson equation self-consistently with the mode space non-equilibrium Green’s function (NEGF) in ballistic limit [34,35,36,37,38,39,40]. The adoption of the vacuum gate dielectric (VGD) paradigm ensures radiation-hardened operation, while the doping-free approach streamlines fabrication and avoids the very high/low temperature-induced dopant detrimental behavior. FET and TFET doping profiles are achieved electrostatically via source and drain doping gates. Our numerical investigation covers potential distribution, transfer characteristics, subthreshold swing, leakage current, on-current, current ratio, and scaling capability. The vacuum devices fulfill the prerequisites for high-performance, radiation-hardened switching applications. Furthermore, our assessment proposes an extrinsic improvement strategy involving doping gate voltage setting to optimize band diagrams and enhance the switching performance of vacuum FET and TFET in the ultrascaled regime. The obtained numerical results underscore the significant potential of the proposed vacuum gate dielectric carbon-based nanotransistors to be a part of the future radiation-immune nanosystems characterized by reconfiguration, high performance, energy-efficient, and radiation hardness. The obtained findings promise to give new impulses to various technological domains, offering solutions that not only enhance reliability but also mitigate the detrimental effects of radiation exposure.

The remaining sections of this paper are structured as follows to provide a comprehensive understanding of the subject matter and its implications. Section 2 will explicitly outline the structural aspects of these novel devices, elucidating their composition and design principles while referring to the relevant references. Building upon this foundation, Section 3 will describe the quantum simulation approach meticulously employed in this computational study. It will delineate the methodologies, algorithms, computational techniques, and theoretical frameworks utilized to numerically model and analyze the behavior of these cutting-edge nanodevices at the atomistic level. Subsequently, Section 4 will comprehensively present and critically discuss the results gleaned from these simulations, shedding light on key observations, trends, and insights garnered throughout the investigation. Finally, Section 5 will serve as a conclusive synthesis, encapsulating the findings, implications, and potential avenues for future research in this particular field.

## 2. Nanodevice Structure

**Figure 1:**
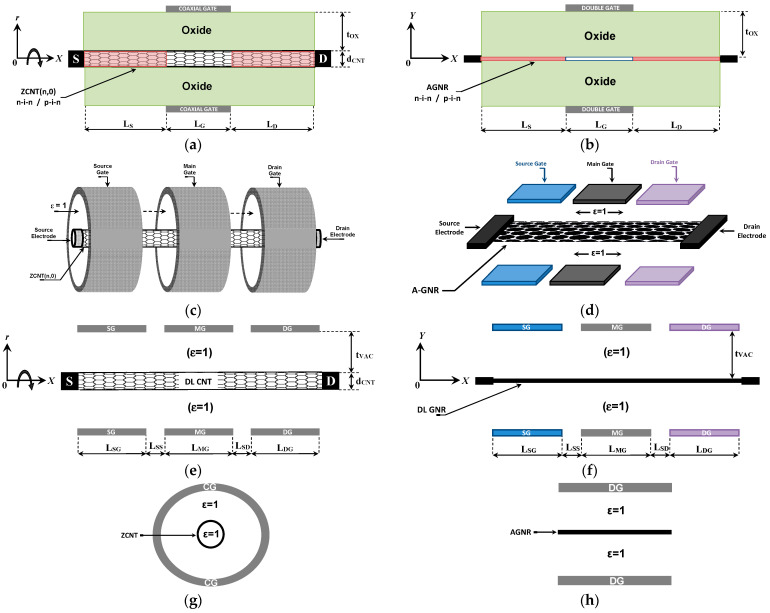
Lengthwise cut views of (**a**) the conventional GAA CNT(T)FET and (**b**) DG GNR(T)FET. Three-dimensional structures of (**c**) the proposed VGD-DLCNT(T)FET and (**d**) VGD-DLGNR(T)FET. Lengthwise cut views of (**e**) the proposed VGD-DLCNT(T)FET and (**f**) VGD-DLGNR(T)FET. Cross-sectional views of (**g**) the proposed VGD-DLCNT(T)FET and (**h**) VGD-DLGNR(T)FET.

Figure 1 illustrates the designs, structures, and 3D perspectives, as well as cross-sectional views, of the proposed and investigated nanotransistors. In fact, carbon nanotubes (CNTs) can be seen as GNRs wrapped around with nanoscale diameters, while GNRs can be produced by slicing graphene sheets into narrow ribbons with nanoscale widths. Both carbon nanotubes and GNRs can display semiconducting or metallic properties. It is worth noting that there are three types of CNTs (zigzag, armchair, and chiral) and two types of graphene nanoribbons (zigzag and armchair). In this computational study, we have employed the (n,0) zigzag CNT type and armchair-edge GNR type due to their suitability in FET applications. Additionally, it is essential to highlight that the channels under consideration are presumed to be flawless, lacking any crystalline defects like stone-wales transformations, edge roughness, or vacancy defects [39,40,41,42,43,44]. In addition to considering the vacuum gate dielectric, monolayer dielectrics can conceptually be adopted to cover the carbon channels, thus avoiding the high radiation-induced trapped charge densities associated with thicker oxide materials [45] and improving the effective coupling capacitance. This enhancement can be particularly significant as the consideration of vacuum gate dielectric alone tends to degrade the coupling capacitance. Note that the impact of channel imperfections can be investigated using advanced quantum computational approaches (e.g., DFT-NEGF-Poisson), which are beyond the scope of this work.

In all nanodevices, zigzag carbon nanotubes (ZCNTs) and armchair-edge graphene nanoribbons (A-GNRs) serve as carbon-based channels for CNT- and GNR-based (T)FETs, as shown in left and right figures, respectively [41,42]. It is worth indicating that coaxial gate configurations (left figures) and double gate configurations (right figures) have been adopted for CNT- and GNR-based devices, respectively [39,40,41,42,43,44]. Note that our study encompasses FET and tunnel FET modes. Additionally, the DL-TFET is not a combination of the Schottky barrier and tunnel FETs because it lacks a Schottky junction between the electrically doped source and the CNT/GNR [46]. Figure 1a,b present lengthwise cut views of the conventional Gate-All-Around (GAA) CNT(T)FET and Double Gate (DG) GNR(T)FET, respectively. These devices feature SiO_2_ gate dielectrics and n-i-n or p-i-n chemical doping profiles [45,47]. The control gate covers the intrinsic channel region for both devices. Figure 1c,d showcase the proposed vacuum gate dielectric doping-free CNT(T)FET and GNR(T)FET, respectively. As their names suggest, these devices operate under dielectric-less [27,28,29] and doping-free [46] paradigms. Electrostatic control is utilized to achieve the doping profile necessary for FET and TFET operation via electrical source and drain doping gates. Figure 1e,f offer lengthwise cut views of the two proposed vacuum devices, revealing the absence of a bulk dielectric material around the channel, which is entirely controlled electrostatically, while 2D dielectrics can conceptually be adopted to cover the carbon channels forming metal-vacuum-2D dielectric-carbon structure, which is beneficial than MOS structure for radiation hardness. Notably, the CNT- and GNR-based vacuum devices are fully reconfigurable and can operate as FETs, TFETs, or BTBT FETs depending on applied biasing conditions (doping and control). Figure 1g,h present cross-sectional views perpendicular to the carbon-based channel. In the case of the CNTFET, even the inner environment of the CNT is considered a vacuum, which offers benefits in terms of immunity against radiation effects. Table 1 provides details of the proposed designs, encompassing configuration, structure, and physical, dimensional, and electrical parameters of the vacuum nanodevices. It is important to note that this information and parameters are nominal, and any changes for parametric investigation’s sake will be explicitly highlighted.

From a fabrication point of view, high-quality carbon nanotubes and graphene nanoribbons can be within reach using advanced bottom-up chemical synthesis approaches. Note that the high quality can be verified by high-resolution imaging [42,43,44]. The doping-free paradigm, together with electrostatic doping, enables the formation of source and drain doping reservoirs without the need for any doping, thus facilitating the relevant fabrication processes [46]. The vacuum gate dielectric, with a given thickness, can be formed via a sacrificial layer deposition and removal process [29]. A gate electrode can be deposited using advanced physical/chemical vapor deposition. Electron beam lithography techniques can be employed to pattern the gate contact, while the etching processes can be then used to precisely define the gate structure and remove unwanted materials. It is worth highlighting that, given the strides in nanotechnology and current advancements in nano-fabrication processes, we believe that the proposed devices are viable [42,43,44].

## 3. Quantum Simulation Approach

Quantum simulations using Non-Equilibrium Green’s Function (NEGF) techniques are crucial for the advancement of nanotransistors, offering essential insights into the intricacies of electron devices on the nanoscale. Via precise simulations of charge carrier behavior, NEGF-based quantum simulations can provide a foundational understanding of how these nanodevices operate, thus guiding their design and optimization processes. A key aspect is accurately predicting and analyzing transport phenomena within nanotransistors, including quantum tunneling effects, carrier scattering mechanisms, and electrostatics. This understanding is vital for addressing challenges related to miniaturization and enhancing device performance. Additionally, NEGF simulations empower researchers to explore innovative device architectures, emerging nanomaterials (as channels, dielectrics, gates, and electrodes), and improved designs, namely carbon nanotransistors utilizing the vacuum gate dielectric paradigm in this case study [34,35,36,37,38,39,40].

**Figure 2:**
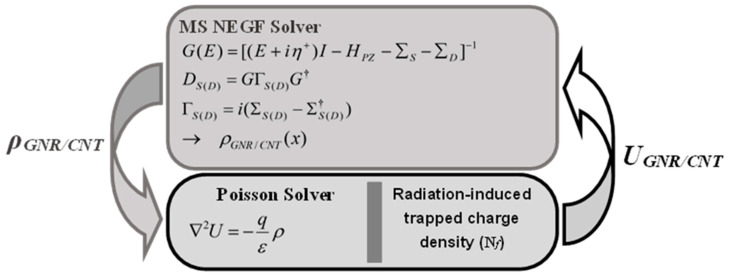
Illustration of the self-consistent procedure in NEGF-based quantum simulation.

Figure 2 illustrates the self-consistent procedure coupling Poisson’s solver with the non-equilibrium Green’s function solver [34,35]. The NEGF formalism employs mode space (MS) representation to optimize computation [36,37]. Only relevant modes are considered in NEGF computations [38,39,40]. Moreover, the assumption of ballistic transport has been made by neglecting the scattering mechanisms (Σ_SCAT_ = 0) arising from the ultra-scaling of the vacuum gate dielectric carbon nanotube/graphene nanoribbon field-effect transistors under investigation [36,37,38,39,40]. The CNT and GNR Hamiltonians utilized in the NEGF-based computational approach are derived from the atomistic nearest-neighbor pz-orbital tight-binding approximation. In other words, only the couplings of p_Z_-orbitals of the CNT and GNR channels have been taken into account [36,37,38,39,40]. The NEGF solver involves computing the retarded Green’s function, G(E), the density of states, D_S(D)_, and the energy level broadening, Γ_S(D)_. These quantities are utilized to calculate the charge density, ρ_GNR/CNT_, which feeds into the Poisson solver [34]. In turn, the Poisson solver provides feedback to the NEGF solver via potential distribution [39,40]. This iterative process continues until convergence or self-consistency is achieved. Subsequently, drain current can be computed from the converged NEGF quantities [34,35,36,37,38,39,40]. Other outputs such as charge density, potential profile, local density of states, energy-position-resolved current spectrum, and band diagrams are also attainable. In brief, the general procedure for the self-consistent simulation can be outlined as follows:

(1) Adjusting the operating bias and providing an initial estimate for the electrostatic potential.
(2) Determining the charge density by solving the NEGF equations.
(3) Utilizing the obtained charge density to solve the Poisson equation and derive a new self-consistent potential to feed the NEGF solver.
(4) Iterating Steps 2 and 3 until achieving self-consistency.
(5) Extracting and computing any device characteristics.

It is worth noting that the radiation-induced trapped charge density, present in conventional CNT/GNR FETs, is integrated into the Poisson solver, as depicted in the same figure [45]. The finite difference method (FDM) is employed to solve the Poisson equation, assuming potential invariance in the width (coaxial) direction in the GNRFET (CNTFET) case [36,37,38]. In the vacuum area, the dielectric constant is set to unity in the finite difference nodes of the Poisson solver. Dirichlet boundary conditions are enforced at all gate contacts, while Neumann boundary conditions are applied to the remaining external ungated boundaries. In previous relevant computational studies, outputs of the quantum simulator have been cross-validated against experimental and advanced computational data, demonstrating good agreement and confirming the accuracy, validity, and predictability of the simulators [45,48]. For further details on the quantum simulation approach employed, readers are referred to classic literature and relevant works [34,35,36,37,38,39,40,45,47,48,49,50,51].

## 4. Results and Discussion

**Figure 3:**
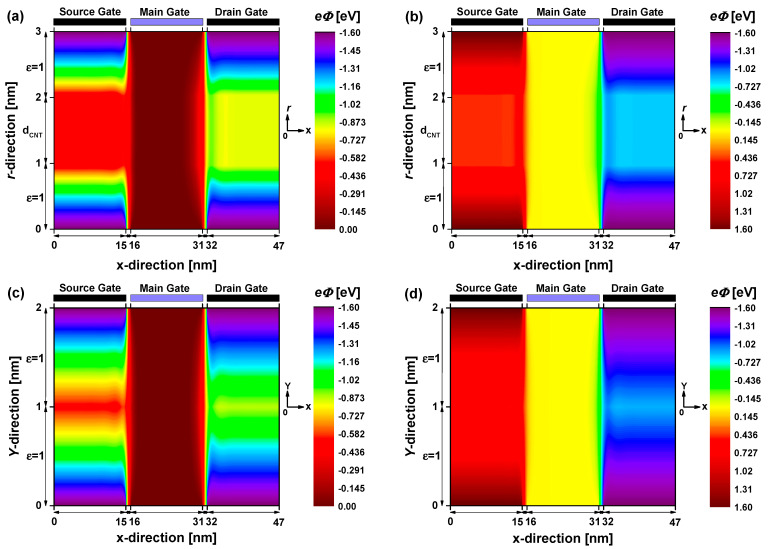
Two-dimensional potential distributions of (**a**) VGD-DLCNTFET, (**b**) VGD-DLCNTTFET, (**c**) VGD-DLGNRFET, and (**d**) VGD-DLGNRTFET.

Figure 3 depicts the two-dimensional electrostatic potential distribution of the proposed vacuum gate dielectric doping-free carbon nanotube/nanoribbon (tunnel) field-effect transistors. Both nanodevices are biased with V_GS_ = 0 V and V_DS_ = 0.4 V. The electrostatic potential, color-mapped after achieving self-consistency, is based on a meshing distance of 1 Å. In all figures, the electrostatic doping effect of the source (drain) auxiliary gate is evident on the left (right) side. Additionally, the electrostatic gating of the middle (main) gate, responsible for controlling the charge carrier, is observable in all nanodevices. In Figure 3a,c, the four corner regions demonstrate similar n-type doping by the two auxiliary gates, forming n-type doped reservoirs akin to conventional MOSFET-like CNT/GNR FETs [52,53]. Conversely, in Figure 3b,d, the p-type (n-type) electrical doping of the source (drain) side, ensured by the negative (positive) applied voltage, is clearly distinguished, forming the required doping profile for tunnel FETs operation. Inspecting the electrostatic figures of the CNTFETs, we can also clearly observe the electrostatic potential corresponding to the empty interior of the CNT, as well as its features within the overall electrostatic potential in comparison to the GNRFET cases.

**Figure 4:**
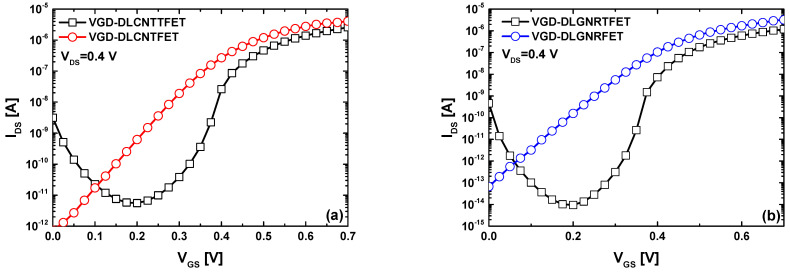
I_DS_-V_GS_ transfer characteristics of (**a**) the VGD-DLCNT(T)FET and (**b**) the VGD-DLGNR(T)FET.

Figure 4 illustrates the I_DS_-V_GS_ transfer characteristics of the VGD-DLCNT(T)FETs and VGD-DLGNR(T)FETs under investigation. It is evident that all vacuum nanoscale devices exhibit normal off-on switching behavior. MOSFET-like transistors demonstrate higher on-state current compared to their TFET counterparts, consistent with their different working mechanisms. Additionally, TFET devices exhibit steeper subthreshold drain currents than MOSFET-like nanodevices. Upon closer inspection, TFET transistors manifest the well-known ambipolar behavior, attributed to the p-type conduction branch due to drain-channel tunneling. The latter starts with gate voltage decreasing when the edge of the valence band underneath the main gate aligns with the edge of the conduction band of the drain, leading to the second type of tunneling mechanism. It is noteworthy that the observed behavior aligns with conventional MOSFET and TFET devices; however, the fully configurable nanodevices studied here are doping-free and dielectric-free. This characteristic offers benefits in terms of fabrication flexibility and radiation immunity, respectively [54,55], making them highly advantageous for radiation-immune nanoelectronics.

In fact, vacuum gate dielectrics offer significant advantages in terms of radiation hardness. However, their effectiveness in controlling carriers is compromised by the low dielectric constant, leading to degraded electrostatics. For this reason, considering 2D dielectrics on carbon channels while maintaining a vacuum environment can offer great benefits. In this context, assessing the subthreshold swing behavior becomes imperative for this performance projection study. It is noted that the subthreshold swing (SS) denotes the alteration in gate voltage necessary to elicit a tenfold augmentation in the drain current of the nanotransistor while it functions in the subthreshold region or beneath the threshold voltage, wherein the transistor demonstrates low drain current and is on the verge of conduction. Numerically, the subthreshold swing can be calculated from the transfer characteristics using the formula [ΔV_GS_/Δlog(I_DS_)].

**Figure 5:**
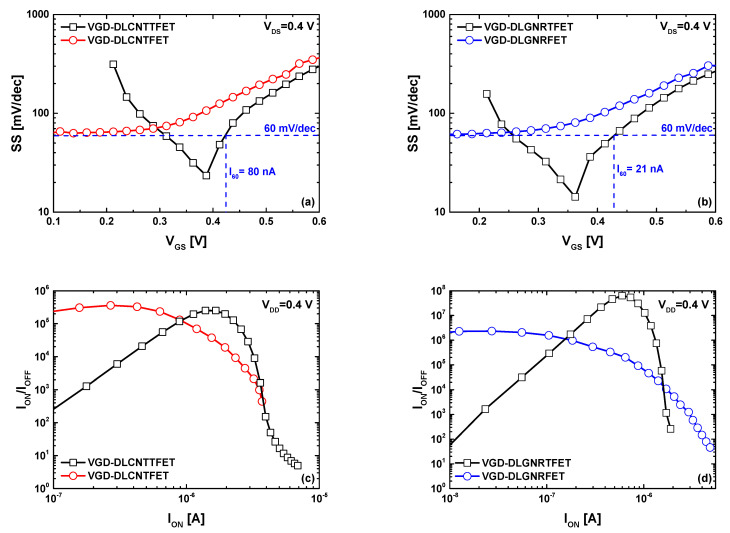
(**a**,**b**) Subthreshold swing versus the gate-to-source voltage, (**c**,**d**) I_ON_/I_OFF_ current ratio versus I_ON_ for VGD-DLCNT(T)FET (**left** figures) and VGD-DLGNR(T)FET (**right** figures).

Figure 5a,b show the subthreshold swing factor as a function of V_GS_ drawn from the I_DS_-V_GS_ transfer characteristics of the VGD-DLCNT(T)FET and VGD-DLGNR(T)FET, respectively. The plots reveal that the best subthreshold swing of MOSFET-like vacuum devices is approximately 60 mV/dec, while the TFET transistors have manifested minimum sub-thermionic subthreshold swing (<60 mV/dec) values of 23.4 mV/dec and 14.3 mV/dec for CNT and GNR-based TFET, respectively. In addition, sub-60 mV/dec values have been recorded for a range of drain currents of TFET devices, which is beneficial for switching applications. Note that the I_60_ metric (i.e., the highest value of drain current that corresponds to 60 mV/dec [56]) is found to be 80 nA (21 nA), recorded approximately at V_GS_= 0.425 V for VGD-DLCNTTFET (VGD-DLGNRTFET). It is to indicate that the I_60_ factor serves as a crucial figure of merit for steep FETs [56]. Figure 5c,d shows the I_ON_/I_OFF_ current ratio as a function of on-state current (I_ON_) considering a power supply voltage (V_DD_) equal to 0.4 V. Note that the two figures are also extracted from the I_DS_-V_GS_ characteristics of the VGD-DLCNT(T)FET and VGD-DLGNR(T)FET considering the aforementioned V_DD_ window. The plots are derived from the transfer proprieties utilizing a window with boundaries set at V_GS−ON_ (right extremity) and V_GS−OFF_ = V_GS−ON_−V_DD_ (left extremity). By shifting the V_DD_ window, we extract I_DS−OFF_ (I_DS−ON_) corresponding to V_GS−OFF_ (V_GS−ON_) at each interval until covering the entire V_GS_ range. Then, the ratios are within reach. It is important to note that this representation enables the visualization of potential current ratios in relation to off- and/or on-current and facilitates the calculation of the maximum reachable current ratio (MRCR). In the CNT-based nanodevices case (Figure 5c), we can see that the two vacuum nanodevices exhibit a maximum reachable current ratio of about ~2 × 10^5^, while in Figure 5d, the recorded MRCR is about ~2 × 10^6^ (~6 × 10^7^) for VGD-DLGNR(T)FET. In both TFET cases, it can be observed that the current ratio decreases as the on-state current decreases. This behavior is attributed to ambipolar behavior, specifically drain-channel tunneling, which is unsuitable for switching applications. Upon comparing the on-state currents corresponding to the maximum reachable current ratio in both bottom figures, it is evident that TFET devices (for both CNT and GNR-based devices) exhibit the highest ones. This is due to their subthermionic subthreshold swing values, which enable low off-state currents and high on-state currents even with low V_DD_ values. The recorded results indicate that the vacuum gate dielectric DLCNT(T)FETs and DLGNR(T)FETs can be reliably operated for radiation-immune switching applications.

**Figure 6:**
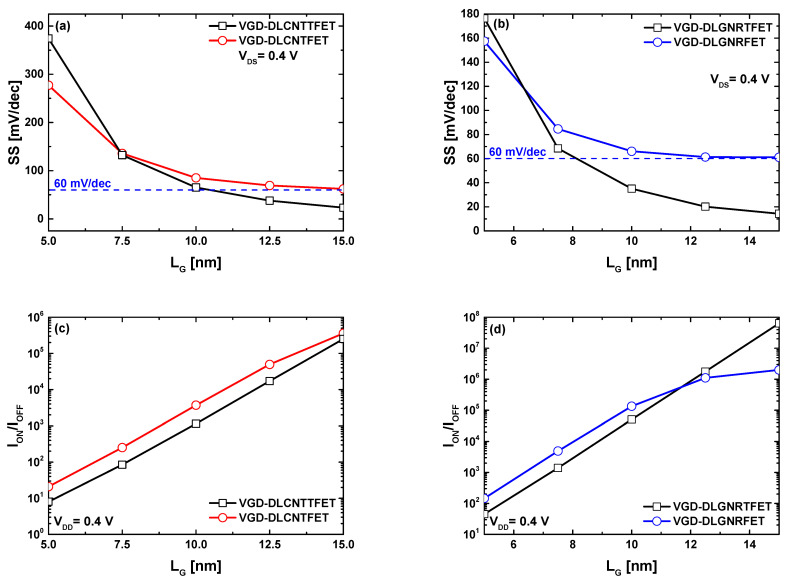
(**a**,**b**) Subthreshold swing; (**c**,**d**) MRCR versus the control gate length for VGD-DLCNT(T)FETs (**left** figures) and VGD-DLGNR(T)FETs (**right** figures).

Figure 6a,b show the behavior of the swing factor as a function of gate length downscaling, considering sub-15 nm gate lengths for VGD-DLCNT(T)FETs and VGD-DLGNR(T)FETs, respectively. As expected, subthermionic subthreshold swing values have been recorded for VGD-DLCNTTFET (VGD-DLGNRTFET) with gate lengths superior then 10.5 (8.2) nm, while in the case of MOSFET-like transistors, the subthreshold swing decreases with gate length increasing to reach the ideal SS value of 60 mV/dec at L_G_ = 15 nm. Inspection of the same figures reveals that at ultrascaled regime (i.e., 5 nm), the MOSFET-like devices exhibit lower SS values than the TFET devices, which is attributed to the high direct source-to-drain tunneling current responsible for the degradation of the TFET subthreshold performance. Figure 6c,d show that the maximum current ratio increases with the gate length increasing for the MOSFET-like and TFET nanodevices, whether in the CNT or GNR case. Note that the MOSFET-like transistor shows a slight superiority in terms of maximum current ratio over its TFET counterpart over the whole considered range in the CNT-based devices case. The same observation has been recorded in the GNR-based devices case. However, the TFETs exhibit greater current ratios for devices with gate lengths greater than 11.7 nm. This behavior can be understood by the steep subthreshold swing (SS) recorded in Figure 6b at the corresponding gate lengths. It is worth noting that similar behavior is expected for CNTTFETs with longer gate lengths. The recorded results indicate that vacuum gate dielectric doping-free carbon nanotube/nanoribbon (tunnel) field-effect transistors have demonstrated a standard scaling capability.

**Figure 7:**
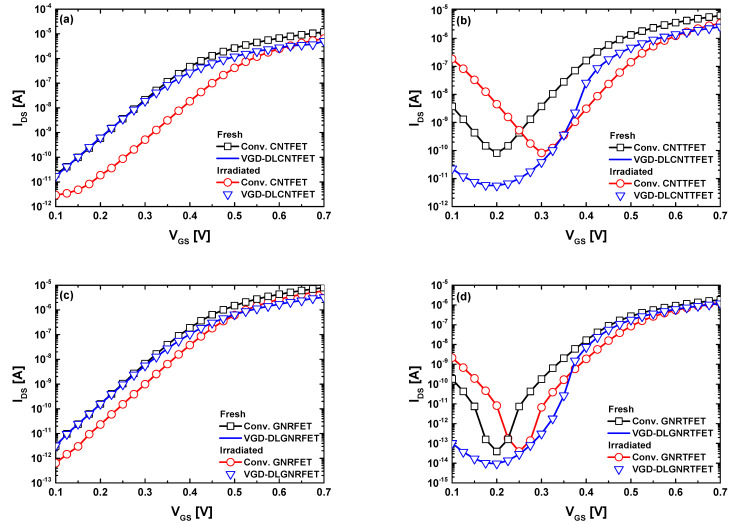
I_DS_-V_GS_ transfer characteristics of (**a**,**b**) the VGD-DLCNT(T)FETs, (**c**,**d**) VGD-DLGNR(T)FETs before and after irradiation, with conventional CNT/GNR-based (T)FETs included for comparison.

Figure 7a,b depict the I_DS_-V_GS_ transfer characteristics of the proposed VGD-DLCNT(T)FETs and conventional CNT(T)FETs before and after irradiation, with the conventional CNT(T)FETs included for benchmarking purposes. It is noteworthy that the conventional designs are assumed to be as described in [57,58], where silicon dioxide (SiO_2_) is typically considered in the baseline CNT-based (T)FETs. It is evident that the proposed vacuum nanodevices exhibit insensitivity to radiation-induced trapped charge density of approximately 4 × 10^12^ cm^−2^ [45,59] owing to the vacuum environment, which is impervious to radiation-induced electrostatics in the nanodevices. Equivalently, there is no radiation-induced generation of electron-hole pairs due to the absence of dielectric, where such generation could occur. However, in the case of conventional CNT(T)FETs, a noticeable shift in threshold voltage (∆V_TH_) toward the positive direction is observed, which may be suitable if the device is operated as a radiation sensor [45,59] but is undesirable when the nanodevice is intended for switching applications [60]. For GNR-based devices, we observe almost identical behavior, as shown in Figure 7c,d, where the proposed designs are immune to radiation-induced threshold voltage shifts, while the conventional designs exhibit sensitivity. Inspecting the recorded shift in GNR-based devices and CNT-based devices, we can observe that the magnitude of the threshold voltage shift is not the same, which may be attributed to the difference in gate configuration (i.e., DG in GNR(T)FET case and GAA in CNT(T)FET case).

Leveraging the doping gates serves not only to embrace the doping-free paradigm and simplify the fabrication process but also to potentially enhance performance. This enhancement can be achieved by adjusting the band diagram to improve transport behavior. In this context, we investigated the possibility of enhancing device performance by adjusting the voltage settings of the doping gates, namely the source gate voltage (V_S_) and drain gate voltage (V_D_).

**Figure 8:**
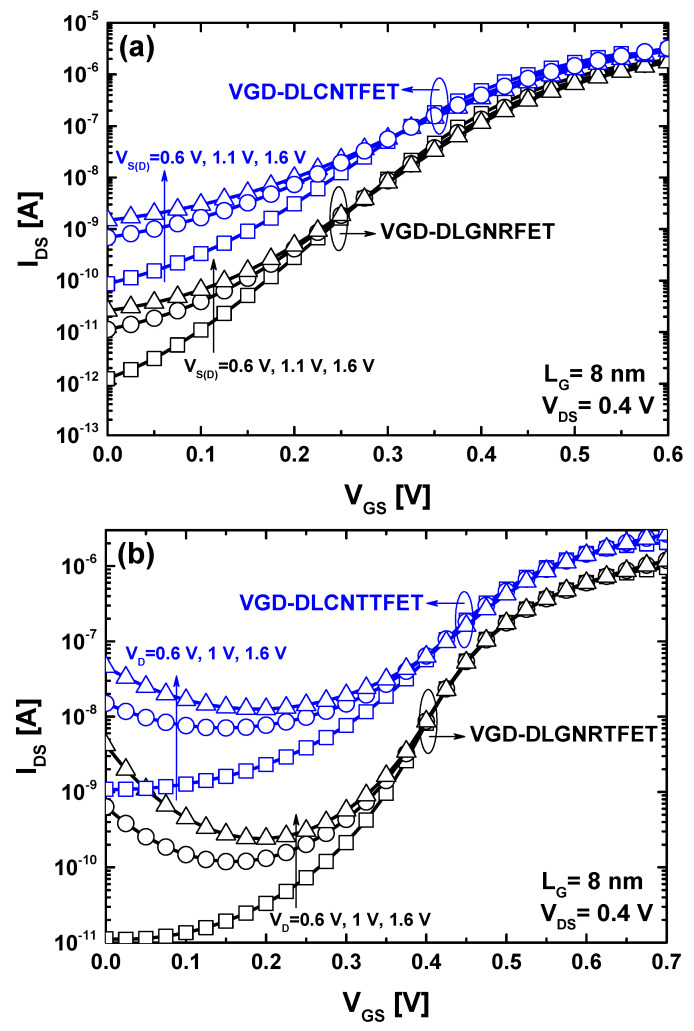
The I_DS_-V_GS_ transfer characteristics of (**a**) MOSFET-like vacuum nanodevices and (**b**) TFET vacuum nanodevices for different electrical doping voltages.

Equivalently, it is about the adjustment of doping electrostatically. As shown in Figure 8a, by decreasing the source and drain doping gates, the leakage current, as well as the subthreshold swing, decreases. Quantitatively, using V_S(D)_ = 1.6 V, The VGD-DLCNTFET (VGD-DLGNRFET) has manifested subthreshold swing and a current ratio of 120.9 mV/dec (78.4 mV/dec) and 426.57 (9.9 × 10^3^), respectively. When using V_S(D)_ = 0.6 V, The VGD-DLCNTFET (VGD-DLGNRFET) has manifested improved subthreshold swing and a current ratio of 80.7 mV/dec (65 mV/dec) and 6.4 × 10^3^ (1.54 × 10^5^), respectively. The same improvements in terms of subthreshold swing and current ratio have been recorded in the TFET case. Quantitatively, using V_S(D)_ = 1.6 V, The VGD-DLCNTTFET (VGD-DLGNTRFET) has manifested subthreshold swing and current ratio of 113.4 mV/dec (59 mV/dec) and 141.78 (2.82 × 10^3^), respectively. When using V_S(D)_ = 0.6 V, The VGD-DLCNTTFET (VGD-DLGNRTFET) has manifested enhanced subthreshold swing and a current ratio of 93.7 mV/dec (50.2 mV/dec) and 632.88 (1.95 × 10^4^), respectively. It should be noted that the improvements observed were recorded for ultra-scaled transistors with an 8-nanometer gate length.

Simulation of such devices and their derivatives (considering different gate configurations) while taking into account scattering mechanisms within the NEGF framework can provide a deeper insight into the behavior and performance projection of carbon-based nanotransistors in radiative environments. Additionally, exploring the analog/RF performance of these devices presents an intriguing avenue for further investigation. Moreover, the study, analysis, and advanced simulation and experimentation on relevant integrated circuits based on vacuum-based transistors, such as static random-access memory (SRAM), microcontrollers (MCUs), field-programmable gate arrays (FPGAs), analog-to-digital converters (ADCs), digital-to-analog converters (DACs), memory modules (Flash Memory, EEPROM), sensor interface ICs, and communication ICs (Transceivers, Modems), offer promising subjects for further research [61,62,63,64,65].

In addition to the aforementioned insights, optimizing vacuum-based devices and electronic systems using bio-inspired optimization approaches (e.g., Ant Colony Optimization (ACO), Genetic Algorithms (GA), Particle Swarm Optimization (PSO), Simulated Annealing (SA)) holds considerable promise [66,67]. Since the vacuum gate dielectric degrades the coupling capacitance, leading to worse carrier control, leveraging negative capacitance ferroelectric materials [68,69,70] to improve such carbon nanodevices can be a matter for further investigation. Furthermore, very interesting results are presented in [71,72,73]. Self-powered application presented in [74,75]. Finally, other directions of research can be found at [76,77,78].

## 5. Conclusions

In this paper, we have computationally proposed vacuum gate dielectric doping-free carbon nanotube/nanoribbon field-effect transistors utilizing a quantum simulation approach. The computational method uses a quantum simulation approach that self-consistently solves the non-equilibrium Green’s function with the electrostatics considering ballistic transport conditions. These novel vacuum nanodevices offer electrostatic reconfigurability, enabling operation in MOSFET-like or TFET-like modes. Our simulations highlight the reliability of the proposed VGD nanotransistors in harsh environments, particularly radioactive mediums, demonstrating high-current ratios, stable threshold voltage, low subthreshold swings, and good scaling capability. Furthermore, we have introduced an effective extrinsic improvement technique involving adjusting the doping gate voltage to enhance transport characteristics via engineered band diagrams. Via rigorous simulation and testing, we have recorded high performance with explicit stability, confirming their immunity to radiation. These compelling numerical results position the proposed VGD carbon transistors as promising candidates for applications in high-energy physics experiments, medical devices, defense systems, aerospace technologies, and other fields where radiation-immune and high-performance nanoelectronics are prerequisites. The unique characteristics of carbon nanotube and graphene nanoribbon field-effect transistors, which involve exhibiting band-to-band tunneling mechanisms in reverse applied gate voltage due to the light effective mass of carriers and a small band gap, present an opportunity for conducting deeper investigations into ultrascaled vacuum gate dielectric BTBT FETs. Such investigations can reveal the capabilities of VGD BTBT devices for high-performance switching and radiation-hardened applications.

## Figures and Tables

**Table 1:** Details of proposed vacuum nanodevice designs.

Parameter	Symbol	VGD-DLGNR(T)FET	VGD-DLCNT(T)FET	Unit
Gate configuration	DG/CG	Double gate	Coaxial gate	-
Dimmer number/chirality	n	13	(13, 0)	-
Gap energy	E_G_	~0.86	~0.81	eV
Width/diameter	W_GNR_/d_CNT_	~1.47	~1	nm
Main Gate length	L_MG_	15	15	nm
S/D gate length	L_S(D)G_	15	15	nm
Spacing	L_SS(D)_	1	1	nm
S/C/D doping (DL)	N_S/C/D_	0	0	nm^−1^
Oxide/vacuum thickness	t_OX_/t_VAC_	1	1	nm
Dielectric constant	ε_VAC_	1	1	-
Temperature	T	300	300	K
Gate-to-source voltage	V_GS_	sweep	sweep	V
Source doping gate voltage	V_S_	1.6 (−1.6)	1.6 (−1.6)	V
Drain doping gate voltage	V_D_	1.6	1.6	V
Drain-to-source voltage	V_DS_	0.4	0.4	V

## Data Availability

The data that support the findings of this study are available from the first corresponding author (K.T.) upon reasonable request.
